# Supplementary material for: Splice-Junction-Based Mapping of Alternative Isoforms in the Human Proteome
Source: Cell Rep. Author manuscript; Available in PMC 2020 Jan 15. (PMC6961840; doi:10.1016/j.celrep.2019.11.026)
Supplement: 3 [file NIHMS1546469-supplement-3.zip › DF2/PXD006675/SmoothMuscleCells-112-P78563-IFSPHEPILEEPADR.pdf]

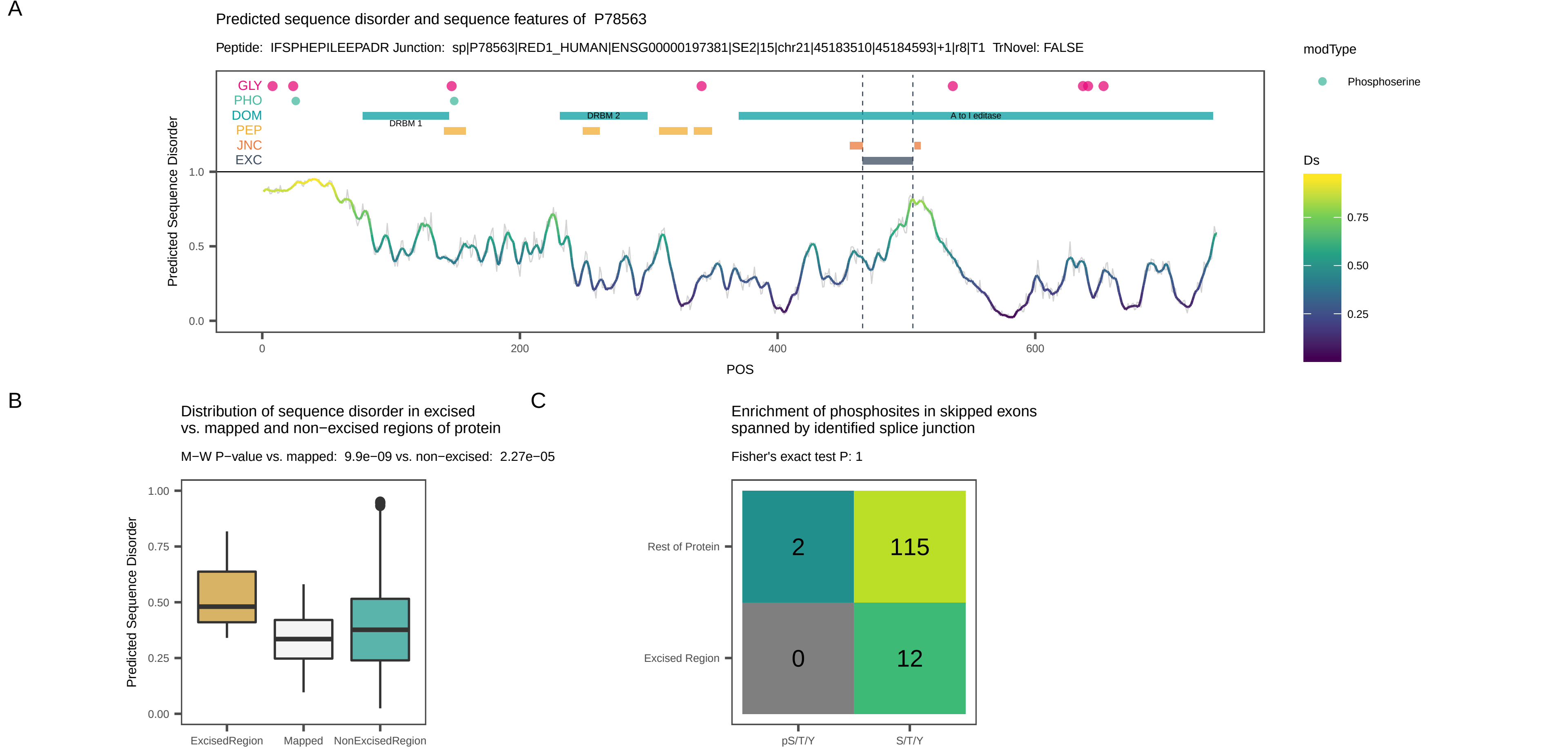
A
Predicted sequence disorder and sequence features of P78563
Peptide: IFSPHEPILEEPADR Junction: sp|P78563|RED1_HUMAN|ENSG00000197381|SE2|15|chr21|45183510|45184593|+1|r8|T1 TrNovel: FALSE
GLY
PHO
DOM
PEP
JNC
EXC
DRBM 1
DRBM 2
A to I editase
Predicted Sequence Disorder
1.0
0.5
0.0
0
200
400
600
POS
modType
Phosphoserine
Ds
0.75
0.50
0.25
B
Distribution of sequence disorder in excised
vs. mapped and non−excised regions of protein
M−W P−value vs. mapped: 9.9e−09 vs. non−excised: 2.27e−05
Predicted Sequence Disorder
1.00
0.75
0.50
0.25
0.00
ExcisedRegion
Mapped
NonExcisedRegion
C
Enrichment of phosphosites in skipped exons
spanned by identified splice junction
Fisher's exact test P: 1
Rest of Protein
Excised Region
2
115
0
12
pS/T/Y
S/T/Y
